# Transgenic Overexpression of Aryl Hydrocarbon Receptor Repressor (AhRR) and AhR-Mediated Induction of CYP1A1, Cytokines, and Acute Toxicity

**DOI:** 10.1289/ehp.1510194

**Published:** 2016-02-05

**Authors:** Christoph F.A. Vogel, W.L. William Chang, Sarah Kado, Kelly McCulloh, Helena Vogel, Dalei Wu, Thomas Haarmann-Stemmann, GuoXiang Yang, Patrick S.C. Leung, Fumio Matsumura, M. Eric Gershwin

**Affiliations:** 1 Department of Environmental Toxicology,; 2 Center for Health and the Environment,; 3 Center for Comparative Medicine, University of California, Davis, Davis, California, USA; 4 Leibniz Research Institute for Environmental Medicine, Düsseldorf, Germany; 5 Division of Rheumatology, Allergy and Clinical Immunology, University of California, Davis, Davis, California, USA

## Abstract

**Background:**

The aryl hydrocarbon receptor repressor (AhRR) is known to repress aryl hydrocarbon receptor (AhR) signaling, but very little is known regarding the role of the AhRR in vivo.

**Objective:**

This study tested the role of AhRR in vivo in AhRR overexpressing mice on molecular and toxic end points mediated through a prototypical AhR ligand.

**Methods:**

We generated AhRR-transgenic mice (AhRR Tg) based on the genetic background of C57BL/6J wild type (wt) mice. We tested the effect of the prototypical AhR ligand 2,3,7,8-tetrachlorodibenzo-p-dioxin (TCDD) on the expression of cytochrome P450 (CYP)1A1 and cytokines in various tissues of mice. We next analyzed the infiltration of immune cells in adipose tissue of mice after treatment with TCDD using flow cytometry.

**Results:**

AhRR Tg mice express significantly higher levels of AhRR compared to wt mice. Activation of AhR by TCDD caused a significant increase of the inflammatory cytokines Interleukin (IL)-1β, IL-6 and IL-10, and CXCL chemokines in white epididymal adipose tissue from both wt and AhRR Tg mice. However, the expression of IL-1β, CXCL2 and CXCL3 were significantly lower in AhRR Tg versus wt mice following TCDD treatment. Exposure to TCDD caused a rapid accumulation of neutrophils and macrophages in white adipose tissue of wt and AhRR Tg mice. Furthermore we found that male AhRR Tg mice were protected from high-dose TCDD-induced lethality associated with a reduced inflammatory response and liver damage as indicated by lower levels of TCDD-induced alanine aminotransferase and hepatic triglycerides. Females from both wt and AhRR Tg mice were less sensitive than male mice to acute toxicity induced by TCDD.

**Conclusion:**

In conclusion, the current study identifies AhRR as a previously uncharacterized regulator of specific inflammatory cytokines, which may protect from acute toxicity induced by TCDD.

**Citation:**

Vogel CF, Chang WL, Kado S, McCulloh K, Vogel H, Wu D, Haarmann-Stemmann T, Yang GX, Leung PS, Matsumura F, Gershwin ME. 2016. Transgenic overexpression of aryl hydrocarbon receptor repressor (AhRR) and AhR-mediated induction of CYP1A1, cytokines, and acute toxicity. Environ Health Perspect 124:1071–1083; http://dx.doi.org/10.1289/ehp.1510194

## Introduction

The function and activity of the aryl hydrocarbon receptor (AhR) is controlled at different levels. In an inactive state the AhR is known to form a complex with heat shock protein (HSP) 90, hepatitis B virus X-associated protein (XAP2) and p23 in the cytosol (Denison and Nagy 2003). Activation of the classical AhR signaling pathway by ligands like 2,3,7,8-tetrachlorodibenzo-*p*-dioxin (TCDD) leads to nuclear translocation of the AhR forming a heterodimer with the AhR nuclear translocator (ARNT) (Reisz-Porszasz et al. 1994). The AhR/ARNT heterodimer binds to dioxin responsive enhancer (DRE) sequences known to induce *Cyp1a1* (cytochrome P450 1a1) and other genes of the AhR gene battery, such as *Cyp1a2*, *Cyp1b1*, and NAD(P)H dehydrogenase [quinone] 1 (*NQO1*) (Okey 2007).

Besides the ligand-dependent activation of AhR, an alternative pathway of AhR signaling has been proposed from studies showing activation and nuclear translocation of the AhR by the second messenger molecule cAMP or forskolin in a protein kinase A- (PKA) dependent manner (Oesch-Bartlomowicz et al. 2005; Vogel et al. 2007b). The ligand-dependent degradation and inactivation of the AhR is processed through the proteasome and other proteases (Davarinos and Pollenz 1999; Morales and Perdew 2007). Another mechanism of AhR control, first reported by Mimura et al. (1999) revealed a new AhR/ARNT- and DRE-regulated gene, known as the AhR repressor (AhRR). From their results, the authors concluded that the AhRR competes with AhR for dimerization with their common partner ARNT, which would inhibit the downstream DNA-binding to DREs and transcriptional activation of genes regulated by the AhR/ARNT dimer. However, studies addressing the role of ARNT for the inhibitory action of AhRR suggested a more complex mechanism than the hypothesized mechanism of negative feedback through sequestration of ARNT to regulate AhR signaling (Haarmann-Stemmann and Abel 2006; Evans et al. 2008; Hahn et al. 2009). Recently, the AhRR has been shown to act as a tumor suppressor gene in several types of cancer cells (Zudaire et al. 2008), which has attracted the interest of an increasing number of cancer scientists. Epigenetic changes of the AhRR have been reported in epidemiological studies and have been associated with exposure to cigarette smoke (Lee et al. 2015; Novakovic et al. 2014; Gao et al. 2015; Fasanelli et al. 2015). Such tumor suppressing actions of AhRR can no longer be explained by the existing theory alone.

Here, we generated the first strain of AhRR overexpressing transgenic B6 mice (AhRR Tg) to investigate if the AhRR is capable of exclusively suppressing the expression of members of the AhR gene battery induced via the classical AhR/ARNT pathway, such as CYP1A1. Furthermore, the effect of the AhRR on TCDD-induced inflammatory genes like cytokines has not been examined. Several reports, including our own work, show that the induction of cytokines, such as interleukin (IL)-6 or IL-8, involves AhR interacting with non-basic helix-loop-helix (bHLH) proteins, such as RelA and RelB, of the NF-κB family through the non-canonical AhR signaling pathway (Vogel et al. 2007b; DiNatale et al. 2010). Interestingly, previous studies showed that the AhRR may interact with non-bHLH proteins [e.g. estrogen receptor α (ERα)] (Kanno et al. 2008).

Although the AhR has an anti-inflammatory role as a mediator of the expression of the immune regulatory enzyme indoleamine 2,3,-dioxygenase (Vogel et al. 2008) and a role in the differentiation of T regulatory cells (Funatake et al. 2005), previous studies have shown that TCDD induces the expression of pro-inflammatory cytokines, such as IL-1β, IL-6, or tumor necrosis factor α (TNFα) (Sutter et al. 1991; Vogel et al. 1997; Rier et al. 2001; Pohjanvirta et al. 2012). In addition, activation of AhR can lead to altered expression of chemokines, including IL-8, as well as CCL and CXCL chemokines (Vogel et al. 2005, 2013; N’Diaye et al. 2006). The role of cytokines in inflammation and carcinogenesis is well established (Lawrence 2007). CXCL chemokines are small cytokine-like proteins and, like cytokines, play an important role in innate and adaptive immune responses (Bonecchi et al. 2009). Chemokines also seem to be critical in inflammatory diseases and cancer progression (Zlotnik 2006; O’Hayre et al. 2008). The current study focused on the analysis of cytokine and chemokine expression in mice exposed to TCDD and examined the role of AhRR in TCDD-mediated toxicity in wt and AhRR Tg mice.

## Materials and Methods

### Cloning of Mouse AhRR cDNA and Preparation of the mAhRR Vector for Microinjection

Total RNA was isolated from murine liver tissue (strain C57BL/6) using trizol reagent (Invitrogen™; ThermoFisher Scientific) and cDNA was synthetized using MMLV reverse transcriptase (Roche Diagnostics USA). Subsequently, the AhRR cDNA was amplified by polymerase chain reaction (PCR) using a proofreading DNA polymerase and the oligonucleotides 5´-GAT​ATC​TGC​AGA​ATT​CCC​ACC​​ATG​ATG​ATT​CCG​TCT​GGA​GAG​TGT​AC-3´ and 5´-TTC​GGG​CCC​AAG​CTT​GGG​TAG​GAA​AAT​TCC​ATC​AGA​GCC-3´ introducing EcoR I /Hind III restriction sites. The cDNA was inserted into pcDNA3.1/myc-His vector (Invitrogen) by homologous recombination using the in-fusion advantage PCR cloning kit (Clontech Laboratories, Inc.). The pcDNA3.1 mAhRR plasmid was digested with *Nae1* and *Apal1* in order to release the 3.6 kb promoter–mmAhrr cDNA-poly A fragment from the vector backbone. The 3.6 kb *Nae1* and *Apal1* fragment was purified from a 0.8% agarose gel. The purified fragment was verified by agarose gel electrophoresis and subsequently purified further on an Elutip column (Whatman® Elutip-d DNA purification minicolumns) according to the manufacturer’s specifications. Following ethanol purification of the fragment, the purified DNA was resuspended into microinjection buffer (5 mM Tris-HCl, 0.1 mM EDTA, pH 7.4, sterile filtered). Eluted purified DNA was subsequently quantified and an aliquot was run on a 0.8% agarose gel to verify its integrity. Prior to pronuclear microinjection, the purified DNA was diluted to a concentration of 2 ng/μl in microinjection buffer.

### Generation and Characterization of AhRR Tg Mice

**Figure 1:**
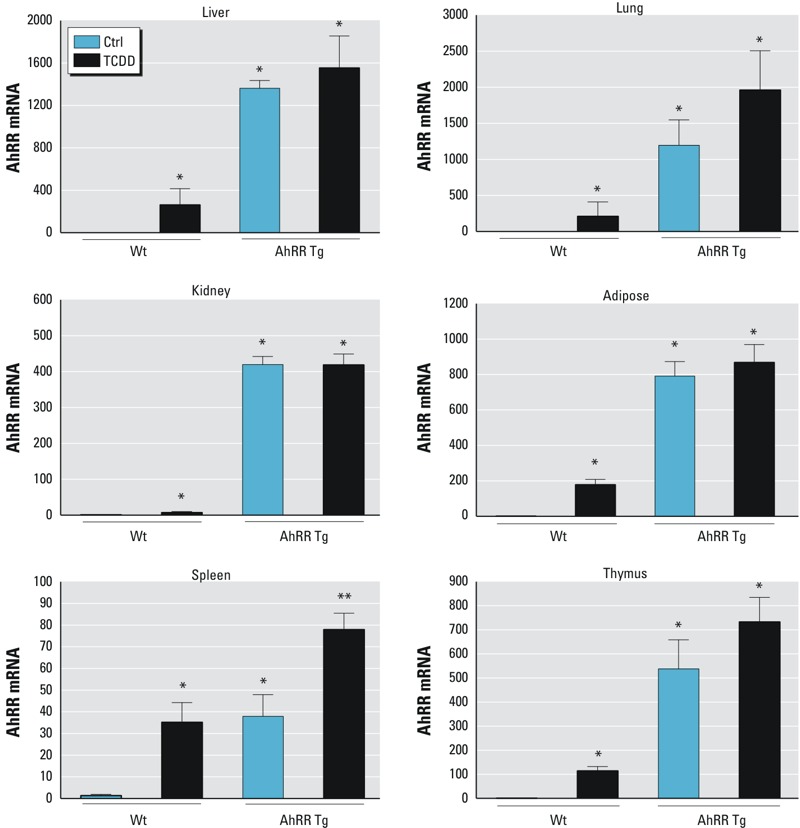
Tissue-level of AhRR expression in response to TCDD in AhRR Tg and C57BL/6J wt mice. Expression of AhRR mRNA in liver, lung, kidney, adipose, spleen, and thymus of C57BL/6 mice wt and AhRR Tg male mice in response to TCDD. Male C57BL/6 wt and AhRR Tg mice were injected i.p. with a single dose of 20 μg/kg TCDD for 24 hr. Control animals received the solvent vehicle. Total RNA from tissues was collected 24 hr post-injection and subjected to qPCR analysis. Values are given as relative units and presented as mean ± SD. *Significantly different from wt control, *p* < 0.05. **Significantly different from AhRR Tg control, *p* < 0.05, by two-tailed Student’s *t*-test.

Pronuclear microinjection of the transgene founder generation and genotyping analysis of tail DNA were performed with the help of the University of California, Davis (UC Davis) Mouse Biology Program (MBP) using standard methods. The 3.6-kb fragment of the pcDNA3.1mmAhRR transgene construct was microinjected into the pronuclei of freshly fertilized oocytes from C57BL/6J mice. Injected oocytes were transferred to day 0.5 postcoitus (dpc) pseudopregnant CD-1/Crl females to generate mmAhrr transgenic mice. Founder transgenic mice were mated to C57BL/6 J mice obtained from Jackson Laboratories; CD-1/Crl mice were obtained from Charles River laboratories. AhRR Tg mice were born in normal Mendelian proportions, grew well, and were fertile. Hematoxylin and eosin (H&E) staining of liver, lung, spleen, thymus and adipose tissue plus lymph nodes revealed no phenotypical differences between wt and AhRR Tg mice (see Figure S1). Four sections of each tissue from six male and six female wt and AhRR Tg mice were evaluated for possible phenotypical differences. All mouse procedures were carried out in accordance with the Institutional Animal Care and Use Committee at UC Davis (IACUC #15723). Tg founder mice were identified by PCR analysis of genomic tail DNA. Tg male founders were screened for the presence of the transgene by PCR analysis of tail-extracted DNA. DNA was extracted from approximately 3 mm tail snips using Qiagen DNeasy blood and tissue kit according to manufacturer’s protocol. DNA was then amplified with a forward primer in the cDNA (5´-CAG​CCC​TGT​CAC​CTG​AAG​AAC​AC-3´) and a reverse primer in the cDNA (5-CGA​CAA​ATG​AAG​CAG​CGT​GTC​AAG-3´) for an expected transgenic amplicon of 385-bp. Twenty-five microliter reactions included 0.4 μM of each primer, 1X PCR buffer, 1.7 mM MgCl_2_, 0.2 mM each dNTPs, 1 Unit Amplitaq polymerase (Applied Biosystem), and 1.3 M Betaine, 1.3% DMSO with approximately 50 ng of template DNA. Thermal cycling included an initial denaturing at 94°C for 5 min; 10 cycles of 94°C for 15 sec, 65°C to 55°C for 30 sec (↓1°C/cycle), 72°C for 40 sec; 30 cycles of 94°C for 15 sec, 55°C for 30 sec, 72°C for 40 sec; final extension of 72°C for 5 min and maintained at 4°C. PCR reactions included a non-template control (NTC), negative wildtype control (B6), and approximately 10 pg plasmid/2 μg genomic DNA as a positive control. PCR Amplicons were sized by agarose gel electrophoresis using a 1 kb+ ladder (Invitrogen). Transgenic offsprings were analyzed for transgene AhRR mRNA and protein expression by quantitative real-time RT-PCR (qPCR) and Western blot analysis, respectively. The founder 6 with an estimated 12 extra copies of transgenic AhRR expressing the highest level of AhRR was selected for further breeding of an AhRR Tg mouse colony. The AhRR Tg mice expressed significantly higher levels of AhRR mRNA in all tissues examined (Figure 1).

### Mice and Treatment

Female and male C57BL/6J wild type (wt), AhRR Tg, and *Ahr* null (AhR^–/–^) mice were housed and treated at UC Davis. AhR^–/–^ mice were a kind gift of Christopher Bradfield (University of Wisconsin). Mice were housed in a selective pathogen-free facility and maintained on a 12:12 hr light/dark cycle and had free access to water and food according to the guidelines set by the University of California. The animals used in this study were treated humanely and with regard for alleviation of suffering. TCDD was administered via a single intraperitoneal (i.p.) injection with 20 μg/kg TCDD for RNA and protein expression analysis according to a previous study (Vogel et al. 2007a). After 24 hr, six animals from each group control and TCDD-treated were killed and their organs were excised, quickly frozen in liquid nitrogen and stored at –80°C for analysis. Epididymal white adipose tissue including the fat tissue only was the source of all adipose tissue used in this study.

### TCDD Toxicity

Male mice were i.p. injected with 50 μg/kg TCDD or the same volume of vehicle alone (corn oil). Six days after TCDD treatment, mice were sacrificed, and alanine aminotransferase (ALT) activity was determined in liver according to the manufacturer’s instructions (Cayman Chemical Company). Changes in levels of ALT and hepatic triglyceride (TRG) were measured in response to 50 μg/kg TCDD, consistent with a previous study (Pande et al. 2005). For mortality studies, female and male mice were treated with a single high dose of 350 μg/kg TCDD (male) or 900 μg/kg TCDD (female) for the indicated period according to a recent study (Pohjanvirta et al. 2012).

### Serum TNFα and IL-1β Determinations

To determine circulating TNF*α* and IL-1*β* levels, ether-anaesthetized mice were bled by retro-orbital sinus puncture at 6 day following 50 μg/kg TCDD injection. Blood was allowed to clot at room temperature, and then centrifuged at 1,000 × g for 15 min. The separated serum was stocked and stored at –80°C until assayed. Concentration of TNF*α* and IL-1*β* was measured using an ELISA kit according to manufacturer’s instructions (R&D Systems Inc.).

### Triglyceride Assay

The method of TRG extraction from mouse liver and TRG analysis were performed according to Butler et al. (1961). In brief, 500 mg liver tissue was homogenized with nine volumes of phosphate buffer in a tissue lyzer. The adsorption of phospholipids with zeolite was followed by the extraction of triglycerides into chloroform. After hydrolysis of triglycerides to fatty acids and glycerol, NaIO_4_ (sodium periodate) was used for oxidation of glycerol to formic acid and formaldehyde. Optical density was determined at 570 nm after formation of a colored complex of formaldehyde and chromotropic acid.

### RNA Isolation and Quantitative Real-Time RT-PCR (qPCR)

The preparation of total RNA and synthesis of cDNA were conducted as described previously (Vogel et al. 2014). qPCR was then performed with the LightCycler® (Roche Diagnostics Corporation) or StepOnePlus™ Real-Time PCR System using the Fast SYBR Green Master Mix (Applied Biosystems™) according to the manufacturer’s protocol. Primer sequences used for qPCR are listed in Table S1. The expression of AhRR, CYP1A1, cytokines, chemokines, as well as TNFα and COX-2 were analyzed. Both, TNFα and COX-2 have been described as targets for TCDD and to be involved in TCDD’s toxicity (Taylor et al. 1992; Vogel et al. 2007a). The data were normalized to the housekeeping gene rps13.

### DNA Promoter Analysis

Gene-based promoter sequences were retrieved from the Ensembl web site (Zerbino et al. 2015). DNA promoter analysis of the cytokine genes was performed using the TFSEARCH program (Heinemeyer et al. 1998).

### Western Blotting

Frozen mouse tissue samples were ground in liquid nitrogen with mortar and pestle. A representative sample of each group was used for Western blot after evaluation of samples from three mice in each group. Representative samples were selected on their average optical density within each group based on visual inspection. The antibodies against actin (sc-1616), CYP1A1 (sc-20772), C/EBPn (sc-150), and NF-κB p65 (sc-372) were purchased from Santa Cruz Biotechnology, while the purified rabbit anti-AhRR antibody was purchased from Novoprotein and NF-κB p105/50 (ab32360) was purchased from abcam.

### Electrophoretic Mobility Shift Assay (EMSA)

Nuclear protein samples were extracted from frozen mouse tissues using methods adopted from a previous report (Vogel et al. 2007b). Oligonucleotide probes containing consensus binding sequences of DRE (5´-GCC​CCG​GAG​TT​G​CGT​GAG​AAG​AGC​CTG​G-3´), C/EBP (5´-TGC​AGA​TTG​CGC​AAT​CTG​CA-3´), and NF-κB (5´-AGC​TTG​CTA​CAA​GGG​ACT​TTC​CGC​TGT​CTA​CTT​T-3´) were synthesized and end-labeled using γ-[^32^P]-ATP (MP Biomedicals) and T4 polynucleotide kinase (EPICENTRE Biotechnologies). EMSA experiments were conducted as previously described (Vogel et al. 2014). In brief, DNA-protein binding reactions were carried out in a total volume of 15 μl containing 10 μg of nuclear protein, 60,000 cpm of double-stranded oligonucleotides, 25 mm Tris buffer (pH 7.5), 50 mm NaCl, 1 mm EDTA, 0.5 mm dithiothreitol, 5% glycerol, and 1 μg of poly(dI·dC). The samples were incubated at room temperature for 20 min. Competition experiments were performed in the presence of a 100-fold molar excess of unlabeled DNA fragments. Protein-DNA complexes were resolved on a 4% nondenaturating polyacrylamide gel and visualized by exposure of the dried gels to x-ray films. Protein-DNA complexes were quantified using a ChemiImager^TM^ 4400 (Alpha Innotech Corp.).

### Isolation and FACS Analysis of Immune Cells from Adipose Tissue

Cells were isolated from epididymal adipose fat pads following a protocol described by Orr et al. (2013). In brief, collagenase digestion was performed with adipose tissue derived from male wt and male AhRR Tg mice treated with 20 μg/kg TCDD for 1, 3, and 6 days. Total live cell counts were obtained by trypan blue exclusion using a hemocytometer. Cell counts of immune cell subsets within isolated adipose tissue cells were evaluated by flow cytometric differential. For immunophenotyping, adipose tissue cells were blocked with anti-mouse CD16/CD32 monoclonal antibody (mAb) and then labeled with fluorophore-conjugated mAbs: F4/80-APC, CD11b-FITC, CD11c-PE-Cy7, Ly-6G-violetFluor 450, and MHC-II (I-A/I-E)-PE. All mAbs were purchased from Tonbo Biosciences. Multi-color flow cytometry was performed using an LSRFortessa cell analyzer (BD Biosciences). Data were analyzed and illustrated using FlosJo software (Tree Star).

### Statistics

Data are expressed as means ± SD. Statistic analyses were performed using Prism software (GraphPad). The comparison between two experimental groups was made using two-tailed Student’s *t*-test for unpaired data. For multiple comparisons, one-way ANOVA with Bonferroni’s test was used. *p-*Value < 0.05 was considered statistically significant.

## Results

### Tissue-level of AhRR Expression in Response to TCDD in AhRR Tg and C57BL/6J Mice

A significantly higher level of AhRR mRNA was detected in all tissues of untreated and TCDD-exposed male AhRR Tg mice relative to untreated C57BL/6J wt mice (Figure 1). The basal level of AhRR mRNA was increased over 1,300-fold (liver and lung), 800-fold (adipose), 400-fold (kidney), 40-fold (spleen), and 550-fold (thymus) in male AhRR Tg mice compared to the corresponding tissues in male wt mice (Figure 1). TCDD significantly induced AhRR mRNA expression in all tissues examined of wt B6 mice including liver, lung, kidney, adipose, spleen and thymus. In AhRR Tg mice, TCDD treatment led to a statistically significant increase in AhRR mRNA (relative to untreated AhRR Tg mice) only in spleen (Figure 1).

### Tissue-specific CYP1A1 Expression in Response to TCDD

**Figure 2:**
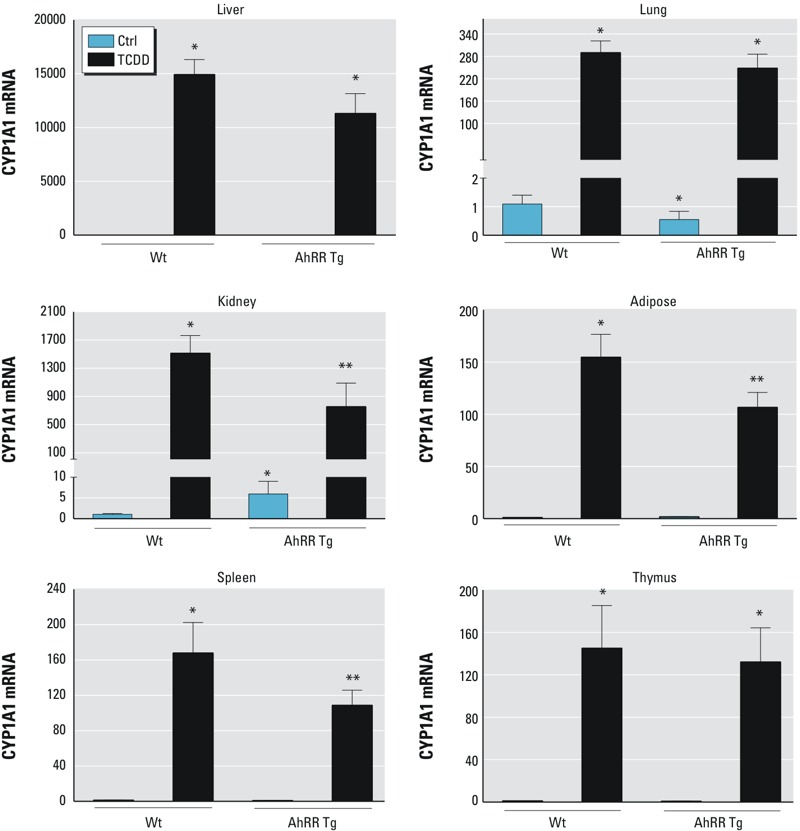
Tissue-level of CYP1A1 expression in response to TCDD in AhRR Tg and C57BL/6J wt mice. Expression of CYP1A1 mRNA in liver, lung, kidney, adipose, spleen, and thymus of C57BL/6 mice wt and AhRR Tg male mice in response to TCDD. Male C57BL/6 wt and AhRR Tg mice were injected i.p. with a single dose of 20 μg/kg TCDD for 24 hr. Control animals received the solvent vehicle. Total RNA from tissues was collected 24 hr post-injection and subjected to qPCR analysis. Values are given as relative units and presented as mean ± SD. *Significantly different from wt control, *p* < 0.05. **Significantly different from wt TCDD, *p* < 0.05, by two-tailed Student’s *t*-test.

**Figure 3:**
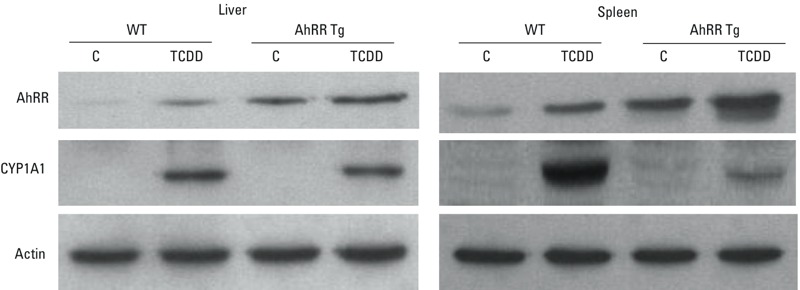
Protein levels of AhRR and CYP1A1 in liver and spleen from male AhRR Tg and C57BL/6J wt mice. Proteins were isolated 24 hr after treatment with vehicle control (C) or 20 μg/kg TCDD and determined by Western blot analysis. Equivalent amounts of whole tissue protein (30 μg of protein) were loaded in each lane on 10% SDS-polyacrylamide gels and analyzed by immunoblotting using AhRR- and CYP1A1 specific antibodies. Bands of Western blot represent replicates from three mice in each group.

In male wt mice, the largest TCDD-induced increase in CYP1A1 mRNA was found in liver (15,000-fold) and kidney (1,500-fold) followed by lung (280-fold), spleen (160-fold), adipose (150-fold), and thymus (140-fold) (Figure 2). The basal level of CYP1A1 mRNA was significantly lower in the lung of male AhRR Tg mice than in wt mice. However, there was no significant difference in CYP1A1 expression in the liver, lung, and thymus between TCDD-treated AhRR Tg and wt mice (Figure 2). Although basal CYP1A1 expression was four times higher in kidney of male AhRR Tg mice compared with male wt mice, TCDD-induced CYP1A1 expression was approximately 50% lower in male AhRR Tg mice than male wt mice. TCDD-induced CYP1A1 expression in spleen and adipose was also significantly lower in AhRR Tg vs. wt mice (approximately 35% and 34%, respectively). The suppression of TCDD-induced CYP1A1 expression in spleen of male AhRR Tg mice vs. TCDD-treated wt mice was confirmed by Western blot (Figure 3). Additionally, the increased expression of AhRR mRNA in TCDD-treated wt mice as well as untreated and TCDD-treated AhRR Tg mice compared with untreated wt mice was confirmed with Western blot analysis using a mouse specific AhRR antibody for mouse AhRR protein as shown in liver and spleen (Figure 3).

### TCDD-induced Expression of Cytokines and Cyclooxygenase-2 (COX-2)

**Figure 4:**
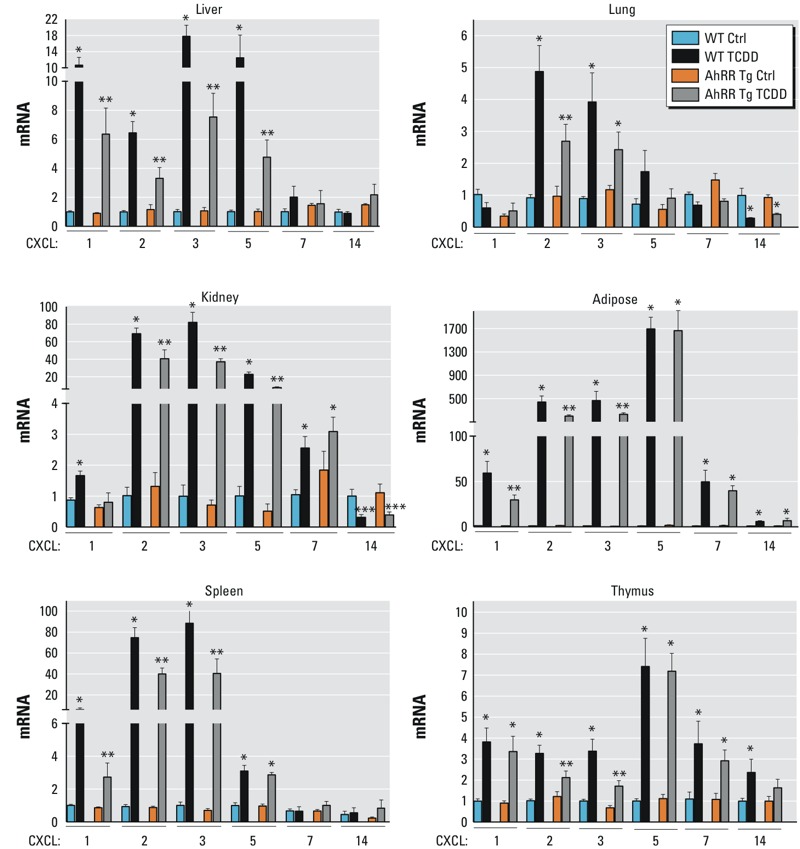
TCDD-mediated induction of CXCL chemokines in AhRR Tg and C57BL/6J wt mice. Expression of CXCL1, CXCL2, CXCL3, CXCL5, CXCL7, and CXCL14 in liver, lung, kidney, adipose, spleen, and thymus of C57BL/6 mice wt and AhRR Tg male mice in response to TCDD. Male C57BL/6 wt and AhRR Tg mice were injected i.p. with a single dose of 20 μg/kg TCDD. Control animals received the solvent vehicle. Total RNA was extracted 24 hr post-injection and subjected to qPCR analysis. Values are given as relative units and presented as mean ± SD. *Significantly different from wt control, *p* < 0.05. **Significantly different from wt TCDD, *p* < 0.05, by two-tailed Student’s *t*-test.

Here we analyzed the expression of TCDD-inducible cytokines including CXCL chemokines CXCL1, CXCL2, CXCL3, CXCL5, CXCL7, CXCL10, and CXCL14. Besides CXCL14 (chromosome 13), the abovementioned CXCL chemokines are located on chromosome 5 of the mouse genome. The promoter regions of the CXCL chemokines contain one or more classical recognition motifs of the AhR/ARNT complex, containing the consensus DRE core sequence 5´-GCGTG-3´ (Table 1). In male wt mice, the largest increases in CXCL chemokines expression in response to TCDD were observed in epididymal white adipose tissue (Figure 4). For example, significant increases in CXCL1 expression in wt mice in response to TCDD were approximately 65-fold in adipose tissue, 11-fold increase in liver, 6.0-fold in spleen, 3.8-fold in thymus, and 1.8-fold in kidney. There was a nonsignificant decrease in CXCL1 expression in lung of TCDD-treated vs. control male wt mice (Figure 4). CXCL2 also was significantly higher in wt mice following treatment with TCDD: approximately 450-fold in adipose, 68-fold in kidney, 74-fold in spleen, 6-fold in liver, and 5- and 3-fold in lung and thymus, respectively. TCDD induced the expression of CXCL3 approximately 500-fold in adipose tissue, 85-fold in spleen, and 18-fold and 82-fold in liver and kidney, respectively, compared to vehicle treated wt mice. The greatest increase in cytokine mRNA in wt mice after TCDD treatment was for CXCL5 (> 1,600-fold in adipose tissue). This was followed by a 20-fold increase in kidney, a 12-fold increase in liver, and 7-fold increase in thymus and approximately 2- to 3-fold increase in lung and spleen of wt mice (Figure 4). The difference in lung CXCL5 expression between TCDD-treated and control wt mice was not significant. CXCL7 mRNA was induced 50-fold in adipose and 2.8-fold in kidney and 3.8-fold in thymus of wt mice. There was no significant difference in CXCL7 expression in spleen between TCDD-treated and control wt mice. The expression of CXCL14 increased significantly only in adipose and thymus of TCDD-treated wt mice. As in wt mice, CXCL14 expression was significantly lower in the lung and kidney of male AhRR Tg mice after TCDD treatment (Figure 4). There was no significant difference in CXCL10 expression between TCDD-treated and untreated mice (AhRR Tg or wt) in any of the tissues tested (data not shown).

**Table 1:** Consensus DRE sites within the promoter region of cytokines. Number and locations of potential consensus DRE sites upstream of the coding sequence of exon 1 on the promoter regions of CXCL chemokines and cytokines.

Gene	No. of DREs	bp upstream of coding sequence
CXCL1	3	–1,305; –309; –290
CXCL2	1	–168
CXCL3	1	–3,236
CXCL5	7	–2,537; –1,830, –1,818; –1,810; –1,802; –1,794; –1,786
CXCL7	1	–2,339
CXCL10	3	–2,509; –2,472; –1,448
CXCL14	1	–30
IL-1β	1	–495
IL-6	1	–398
IL-10	1	–335
IL-22	1	–1,082
DRE consensus sites within the promoter region of the cytokines were analyzed using the TFSEARCH program (Heinemeyer et al. 1998). Gene-based promoter sequences were retrieved from the Ensembl web site (Zerbino et al. 2015).		

In comparison to wt mice, TCDD-treated AhRR Tg mice had significantly lower expression of CXCL2 and CXCL3 (Figure 4). This was true for all tissues with the exception of CXCL3 in the lung. In AhRR Tg mice, CXCL3 expression was significantly lower in TCDD-treated mice compared with TCDD-treated wt mice in all tissues except the lung, with the greatest difference in expression (approximately 50%) in thymus and kidney. CXCL5 expression in the kidney and liver was significantly lower in TCDD-treated AhRR Tg mice than in TCDD-treated wt mice.

**Figure 5:**
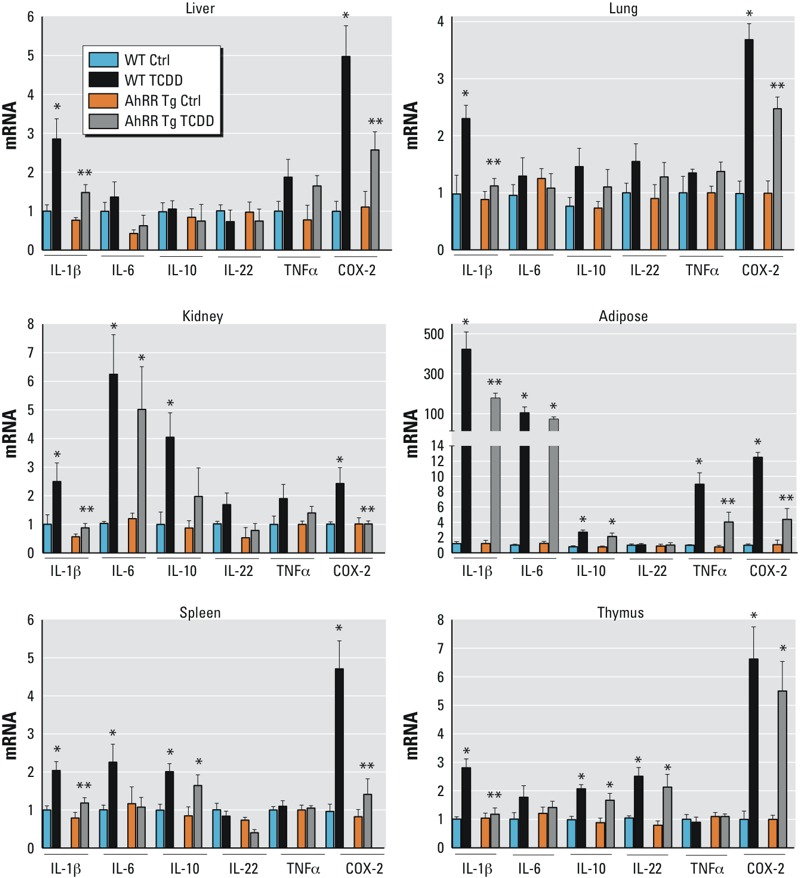
TCDD-mediated induction of cytokines and COX-2 in AhRR Tg and C57BL/6J wt mice. Expression of IL-1β, IL-6, IL-10, IL-22, TNFα, and COX-2 in liver, lung, kidney, adipose, spleen, and thymus of C57BL/6 mice wt and AhRR Tg male mice in response to TCDD. Male C57BL/6 wt and AhRR Tg mice were injected i.p. with a single dose of 20 μg/kg TCDD. Control animals received the solvent vehicle. Total RNA was extracted 24 hr post-injection and subjected to qPCR analysis. Values are given as relative units and presented as mean ± SD. *Significantly different from wt control, *p* < 0.05. **Significantly different from wt TCDD, *p* < 0.05, by two-tailed Student’s *t*-test.

Cytokines, such as IL-1β, IL-6, IL-10, and IL-22 are target genes of the AhR *in vitro* as well as *in vivo* (Sutter et al. 1991; Pande et al. 2005; Marshall et al. 2008; Bankoti et al. 2010; DiNatale et al. 2010; Pohjanvirta et al. 2012; Rohlman et al. 2012; Vogel et al. 2013; Kennedy et al. 2014). Promoter sequence analysis revealed that each of the abovementioned cytokines contain a potential consensus DRE binding site upstream of their coding region (Table 1). Similar to the results from chemokines, the largest increase of the cytokines IL-1β (422-fold) and IL-6 (100-fold) was found in adipose tissue of wt mice treated with TCDD for 24 hr (Figure 5). TCDD treatment induced IL-1β in all tissues tested of wt mice, but not in lung, kidney, spleen or thymus derived from male AhRR Tg mice. As shown in Figure 5, the expression of IL-6 in the spleen was significantly higher in TCDD vs. control wt mice, while there was no significant difference between IL-6 expression in TCDD AhRR Tg compared with control wt mice. The expression of IL-10 mRNA was 4.0-fold and 3.0-fold increased in kidney and adipose, respectively, and 2.0-fold in thymus and spleen of male TCDD-treated wt vs. control wt mice. A statistically significant 2.5-fold increase of IL-22 was found in thymus of TCDD-treated mice, but not in any other tissue examined (Figure 5).

In addition, we measured the expression of TNFα and COX-2. A significant increase of TNFα by about 9-fold and 4-fold was found in adipose tissue of TCDD-treated male wt and AhRR Tg mice, respectively, compared with untreated mice (Figure 5). COX-2 was induced by TCDD in all tissues of wt mice. There was no significant difference in COX-2 expression in the kidney and spleen of AhRR Tg mice according to TCDD treatment. Again, the highest increase was found in adipose of about 12-fold and 5-fold in wt and AhRR Tg mice, respectively. Except for thymus, the TCDD-induced expression of COX-2 was lower in tissues from AhRR Tg mice compared to wt mice (Figure 5). As an overview of the results, data of gene expression changes shown in Figures 1 to 5 are summarized in Table S2. TCDD-induced expression of the cytokines as well as COX-2 was AhR-dependent because TCDD had no significant effect on the expression of cytokines or COX-2 in tissues from male AhR^–/–^ mice as shown for adipose tissue (see Figure S2). No significant change of cytokine and COX-2 expression was found in other tissues including liver, lung, kidney, spleen and thymus of TCDD-treated vs. control AhR^–/–^ mice (data not shown).

In order to examine gender-specific effects, we treated female wt and female AhRR Tg mice with TCDD and analyzed the expression of AhRR, CYP1A1, and cytokines 24 hr after treatment in parallel with male mice. The results showed no significant difference of AhRR and CYP1A1 mRNA in control and TCDD-treated female wt and AhRR Tg mice compared to the corresponding male mice (Figures 1 and 2; see also Figure S3). On the other hand, CXCL1 and CXCL2 expression was significantly lower in spleen and adipose from TCDD-treated female wt and AhRR Tg mice compared with TCDD-treated male wt and AhRR Tg mice (Figure 4; see also Figure S3). There was no significant difference in IL-1β and IL-6 expression in the spleen of TCDD vs. untreated female wt and AhRR Tg mice. IL-1β and IL-6 expression increased significantly in adipose tissue of TCDD vs. untreated female wt and AhRR Tg mice, but levels were significantly lower than those observed in TCDD-treated male wt and AhRR Tg mice (Figure 5; see also Figure S3).

### Accumulation of CD11b and F4/80 Cells in Adipose Tissue Following TCDD

**Figure 6:**
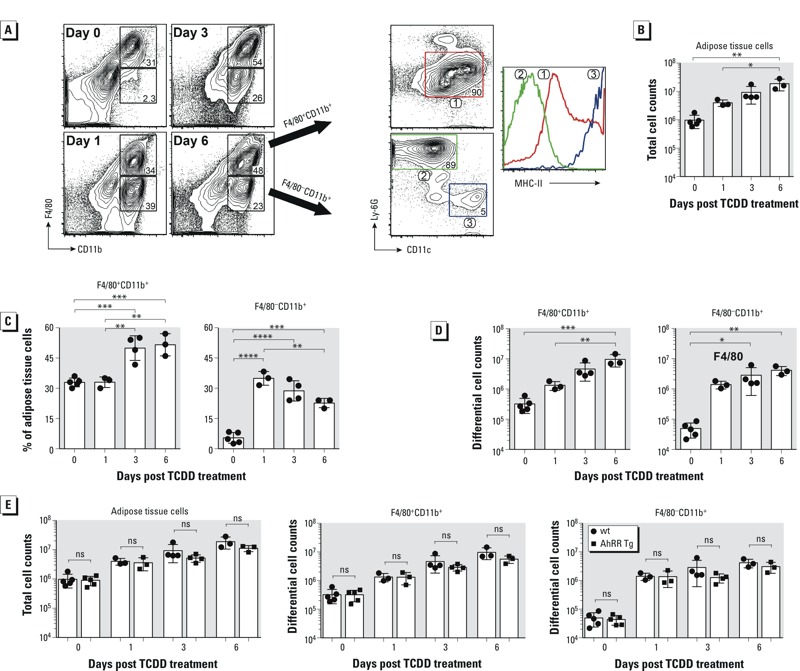
Accumulation of F4/80^+^CD11b^+^ and F4/80^–^CD11b^+^ cells in adipose tissue after TCDD exposure. Enhanced accumulation of F4/80^+^CD11b^+^ and F4/80^–^CD11b^+^ cells in adipose tissue after TCDD exposure. Adipose tissue cells were isolated from wt and AhRR Tg mice treated with 20 μg/kg TCDD for 1, 3, and 6 days or 0 day (untreated controls). (*A*) Shown in left panels are representative FACS plots for the expression of F4/80 and CD11b after gating on live cells. Numbers within boxes represent percentages of the respective cell populations among total gated cells. Three cell subsets were identified based on the expression of CD11c and Ly-6G (right panels), and the overlaid histogram of MHC-II on these cells is shown. (*B*) Total cell number isolated from adipose tissues of TCDD treated wt mice. (*C*) Percentage and (*D*) differential cell number of F4/80^+^CD11b^+^ and F4/80^–^CD11b^+^ cell subsets in adipose tissues of wt mice after TCDD treatment. (*E*) Comparison of total adipose cell and differential cell subset numbers in adipose tissues of wt and AhRR Tg mice after TCDD treatment. Shown are results from three to five individual mice per group. ns, not significant by two-tailed Student’s *t*-test. *p < 0.05. **p < 0.01. ***p < 0.001. ****p < 0.0001

To test whether TCDD would stimulate infiltration of immune cells into adipose tissue, we isolated adipose tissue cells from epididymal adipose tissue of male wt and AhRR Tg mice and assessed their phenotype by flow cytometry. Fluorescence-activated cell sorting (FACS) analyses indicated a rapid increase of F4/80^–^CD11b^+^ cell subset 1 day post TCDD treatment in male wt mice (Figure 6A). This cell subset is composed of two major cell types: CD11c^–^Ly-6G^+^MHC-II^–^ neutrophils and CD11c^+^Ly-6G^–^MHC-II^hi^ dendritic cells. Although frequencies of F4/80^–^CD11b^+^ cells gradually declined (Figure 6C), their numbers remained significantly higher than those in untreated mice (Figure 6D), as a result of continuous accumulation of immune cells in adipose tissues induced by TCDD (Figure 6B). The F4/80^+^CD11b^+^ cell subset was phenotypically more homogenous (CD11c^±^Ly-6G^–^MHC-II^int/hi^), in contrast to the F4/80^–^CD11b^+^ cell subset (Figure 6A, left panel). The phenotype of F4/80^+^CD11b^+^ cells resembles inflammatory macrophages and became the dominant cell population 3 days post TCDD treatment (Figure 6C). On day 6, the frequencies of F4/80^+^CD11b^+^ cells stayed at the peak level (Figure 6C) and the cell number of this cell subset continued to rise (Figure 6D). The number of F4/80^+^CD11b^+^ and F4/80^–^CD11b^+^ cells in adipose tissues of wt mice were marginally higher than those of AhRR Tg mice on day 3 and 6 post TCDD treatment (Figure 6E, middle and right panels). The differences were resulting from slight increases of cell accumulation in adipose of wt mice (Figure 6E, left panel). For the frequencies of adipose tissue F4/80+CD11b+ or F4/80-CD11b+ cell subsets, there were no differences between those of wt and AhRR tg mice (data not shown).

### DNA Binding Activity of TCDD-Sensitive Transcription Factors in wt and AhRR Tg Mice

**Figure 7:**
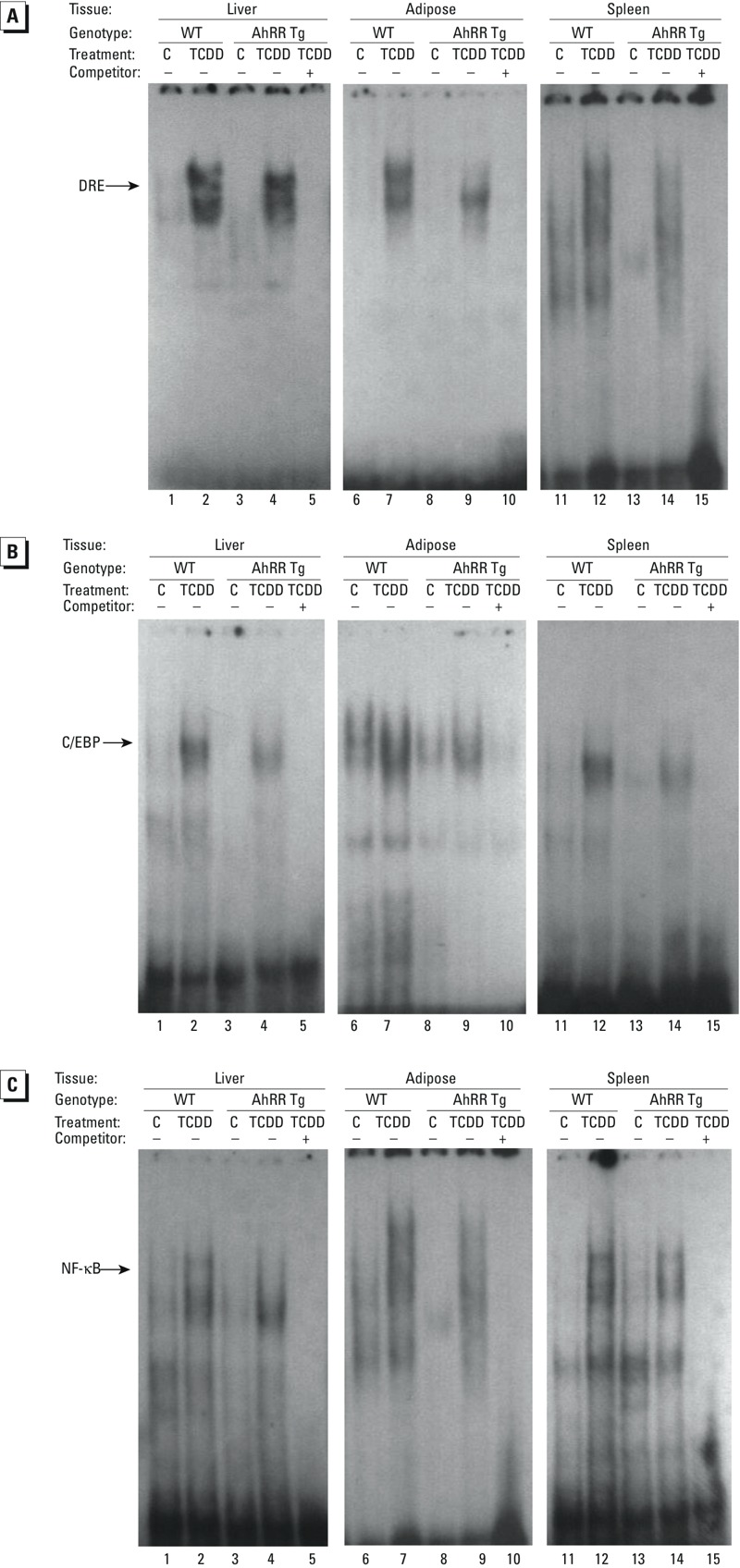
DNA binding activity of DRE, C/EBP and NF-κB complexes in AhRR Tg and C57BL/6J wt mice. DNA binding activity to 32P-end-labeled oligonucleotides containing (*A*) DRE, (*B*) C/EBP and (*C*) NF-κB consensus elements. Male C57BL/6 wt and AhRR Tg mice were injected i.p. with a single dose of vehicle control (lanes 1, 3, 6, 8, 11, and 13,) or 20 μg/kg TCDD (lanes 2, 4, 7, 9, 12, and 14). Nuclear proteins were extracted 24 hr post-injection. For specificity a 200-fold molar excess of unlabeled probe was added as competitor (lanes 5, 10, and 15).

**Figure 8:**
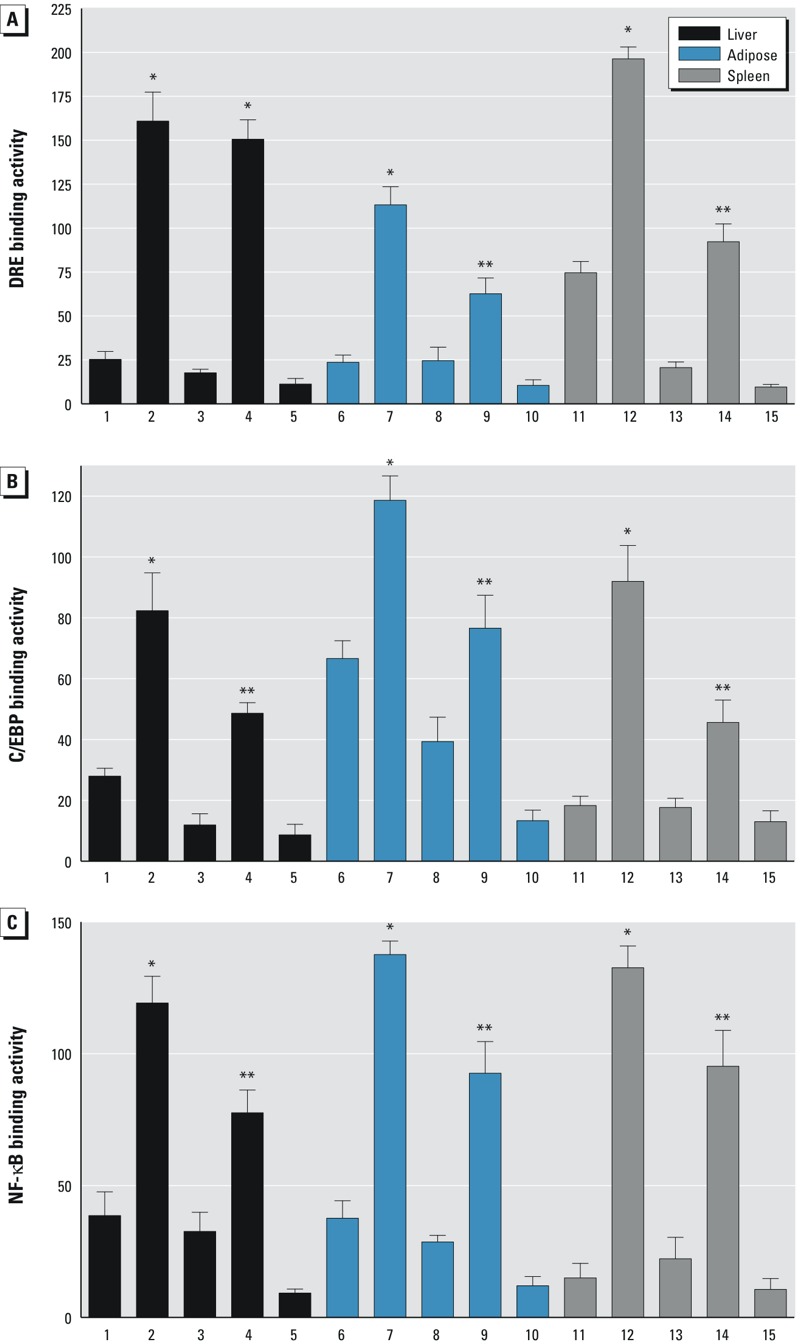
Densitometric evaluation of band intensities of the DNA binding complexes. Band intensities of DNA binding complexes of nuclear proteins to (*A*) DRE (*B*) C/EBP, and (*C*) NF-κB consensus elements are shown as densitometry data. Numbers on the *x*-axes correspond to the lane numbers shown in Figure 7. Averages for each tissue from three different mice are shown as mean values ± SD. *Significantly different from wt control, *p* < 0.05. **Significantly different from wt TCDD, *p* < 0.05, by two-tailed Student’s *t*-test.

**Figure 9:**
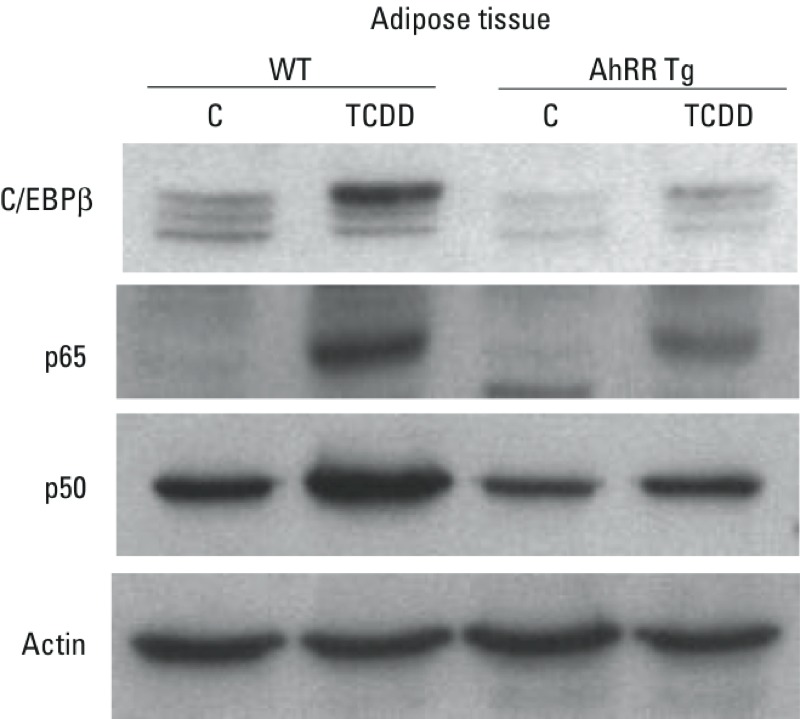
Protein levels of C/EBPβ, NF-κB p65, and p50 in nuclear extracts from adipose tissue. Nuclear protein extracts were prepared from epididymal adipose tissue derived from male wt and AhRR Tg mice. Mice were treated for 24 hr with corn oil as vehicle control (C) or 20 μg/kg TCDD and protein levels determined by Western blot analysis. Equivalent amounts of nuclear protein (15 μg of protein) were loaded in each lane on 10% SDS-polyacrylamide gels and analyzed by immunoblotting using C/EBPβ, NF-κB p65, and p50 specific antibodies. A representative gel image of each tissue sample from three mice of each group is shown.

Sequence analysis for potential transcription factor binding sites of the up-stream regulatory regions of the chemokines and cytokines investigated revealed potential consensus DRE binding sites for the AhR/ARNT complex on their promoter region (Table 1). Besides consensus DRE sites, DNA binding elements for CCAAT/enhancer binding protein (C/EBP) and NF-κB are particularly important activators of transcriptional regulation of cytokines, such as IL-1β or IL-6 (Xiao et al. 2004; Basak et al. 2005; Bonecchi et al. 2009). Previous reports have shown that TCDD and the AhR may affect the binding activity of C/EBP and NF-κB (Vogel et al. 2004; Puga et al. 2000). Therefore, the effect of TCDD on consensus binding sites for C/EBP and NF-κB was tested, and DNA binding activity in liver, spleen and adipose from male wt mice was compared with the corresponding tissue from male AhRR Tg mice. The TCDD-induced DRE binding activity was comparable in nuclear extracts from liver derived from wt and AhRR Tg mice (Figure 7A). However, a lower DRE binding activity was found in nuclear extracts from adipose as well as spleen of TCDD-treated AhRR Tg mice compared with the nuclear extracts from TCDD-treated wt mice (Figures 7A and 8A). On the other hand, the DNA binding activity of C/EBP and NF-κB in liver, adipose, and spleen was lower in TCDD-treated AhRR Tg mice compared with TCDD-treated wt mice, consistent with overexpression of AhRR in the Tg mice (Figures 7 and 8). Western blot analysis suggested that the transcription factors C/EBPβ, NF-κB p50 and p65 accumulated in the nuclear protein fraction of adipose tissue after TCDD treatment for 24 hr, and that nuclear accumulation of all three proteins was more pronounced in adipose from wt mice compared with AhRR Tg mice (Figure 9). A representative sample of each group was selected after evaluation of the tissue samples from three mice in each group.

### TCDD-induced Acute Toxicity in AhRR Tg and C57BL/6J Mice

**Figure 10:**
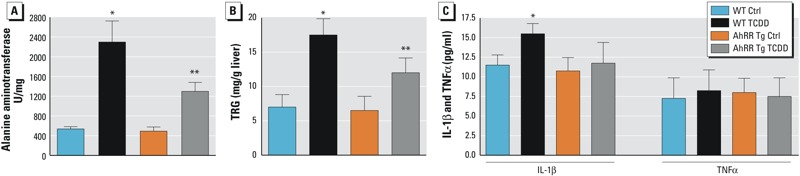
Effect of TCDD on levels of ALT and TRG in liver and serum levels of IL-1β and TNFα in AhRR Tg and C57BL/6J wt mice. (*A*) Effect of TCDD on alanine aminotransferase (ALT) levels in liver, (*B*) Hepatic triglyceride (TRG) levels, and (*C*) Serum levels of IL-1β and TNFα in male wt and AhRR Tg mice treated with corn oil (Control) or 50 μg/kg TCDD for 6 days. Six animals (12 weeks of age) were included in each group. Data are presented as mean ± SD. *Significantly different from wt control, *p* < 0.05. **Significantly different from wt TCDD, *p* < 0.05, by two-tailed Student’s *t*-test.

**Figure 11:**
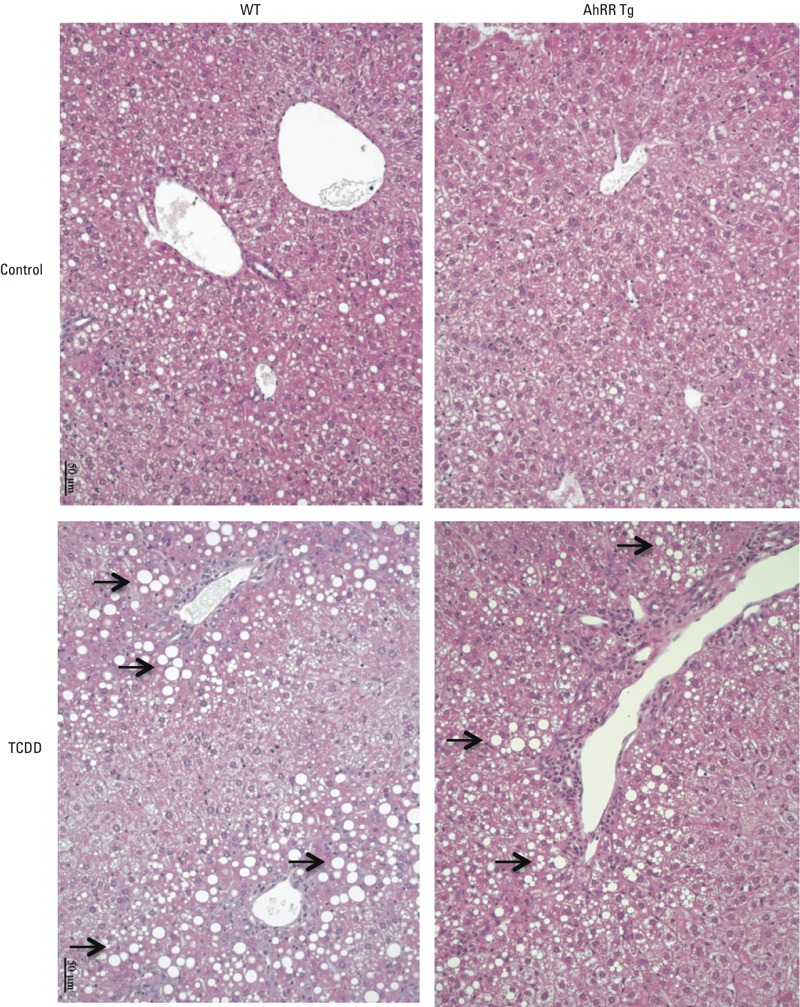
Hematoxylin and eosin stained liver sections of male wt and AhRR Tg mice. Liver sections were prepared and stained following a single i.p. dose of 50 μg/kg TCDD for 6 days. The arrows indicate clear vacuoles caused by histological fixation dissolving the accumulated lipids. Images represent replicates from three mice in each group.

Total liver ALT activity was found significantly increased by TCDD at day 6 in both, wt and AhRR Tg male mice. We observed a more than 4-fold increase of ALT in liver of wt mice (Figure 10A); in contrast, the elevation in liver ALT was only 2-fold in AhRR Tg mice exposed to TCDD. TRG was significantly higher in liver of TCDD wt mice compared with untreated wt mice (Figure 10B). TRG was significantly lower in TCDD-treated AhRR Tg mice compared with TCDD wt mice. In addition to increases in mean hepatic ALT and TRG levels, IL-1β was also significantly increased in serum of TCDD-treated vs. untreated mice (Figure 10C). The serum level of TNFα was below 10 pg/mL and did not change by TCDD. Hepatic TRG and serum IL-1β levels in TCDD-treated AhRR Tg mice were significantly lower than levels in TCDD-treated wt mice. TCDD also induced a steatotic phenotype as shown in H&E stained liver sections from wt male mice after TCDD exposure (Figure 11) as reported earlier (Angrish et al. 2011). H&E stained sections from livers of TCDD-treated mice show an increasing degree of steatosis represented by the clear vacuoles caused by histological fixation dissolving the accumulated lipids.

**Figure 12:**
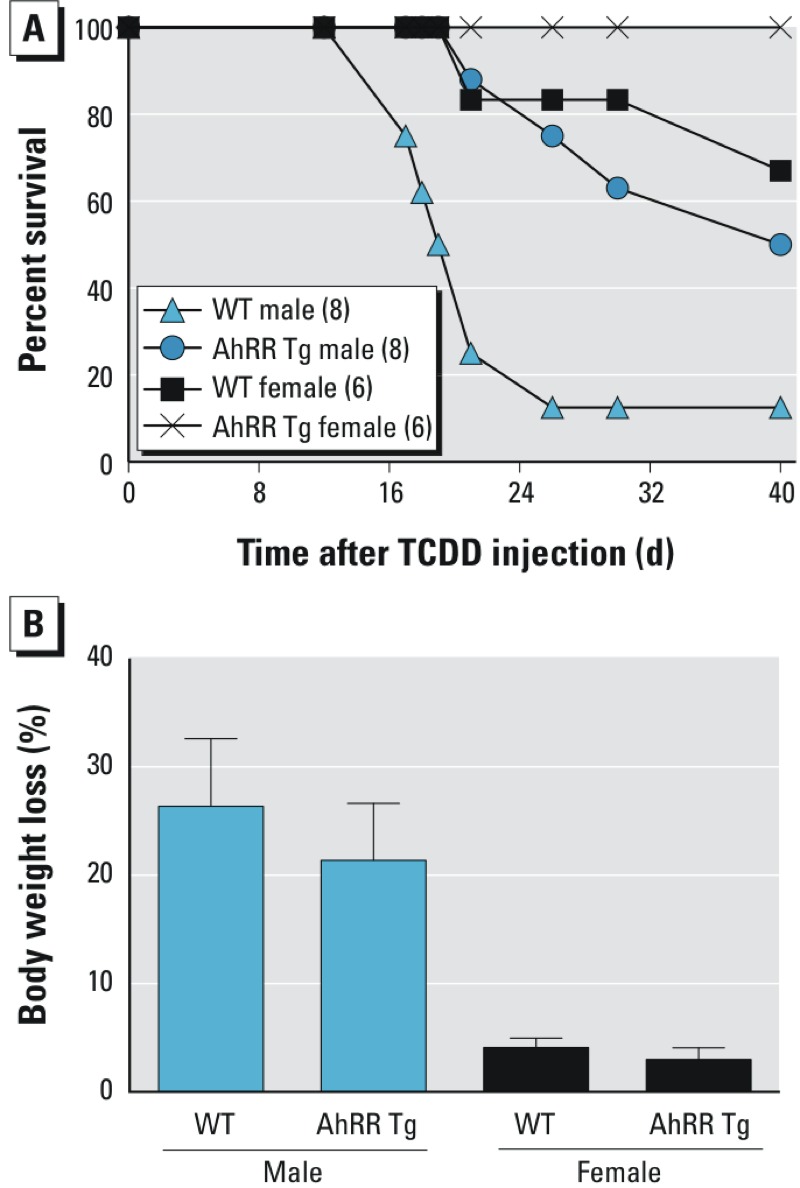
TCDD-mediated lethality in AhRR Tg and C57BL/6J wt mice. (*A*) Percent survival and (*B*) bodyweight loss of male and female wt and AhRR Tg mice following a single i.p. dose of 350 μg/kg TCDD for males and 900 μg/kg TCDD for females. Eight male and six female mice (12 weeks of age) were included in each group. The values for body weight are depicted in percent loss of total body weight taken 17 days after initial injection of TCDD. Blue bars, male TCDD-treated mice; black bars, female TCDD-treated mice.

Moreover, TCDD-treated male AhRR Tg mice revealed reduced acute lethal toxicity compared to their corresponding male wt mice. As shown in Figure 12A, a high dose of 350 μg/kg TCDD was lethal for about 85% of male wt mice between day 17 and day 20 post-injection. On the other hand, none of the male AhRR Tg mice died with this dose before day 21 post-injection. Only 40% of male AhRR Tg mice died before day 30 post-TCDD injection. Male wt and AhRR Tg mice had reduced body weight 17 days after TCDD injection (approximately 26.4% in wt and 21.4% in AhRR Tg mice) (Figure 12B). In contrast, female wt and female AhRR Tg mice lost less than 5% of their body weight after TCDD injection. Two female wt mice died with a dose of 900 μg/kg TCDD at day 22 and day 39 after TCDD injection (Figure 12B). None of the female AhRR Tg mice died after the 900 μg/kg dose of TCDD until the end of the observation period of 90 days.

## Discussion

The current study shows that overexpression of AhRR in transgenic mice may suppress the induction of the prototypical AhR-regulated gene CYP1A1 in certain tissues such as kidney, spleen, and adipose. Reduced CYP1A1 expression in response to TCDD is consistent with an interaction of AhRR with the classical AhR/ARNT complex. A recent study with keratinocytes showed that the repressed CYP1 activity is not related to the expression level of AhRR (Tigges et al. 2013). The mechanism, however, for the tissue-specific effect of AhRR overexpression on CYP1A1 induction is unclear. Since AhRR may dimerize with ARNT, which leads to competition with AhR for binding to DRE response elements, a limited protein pool of ARNT in certain tissues could explain the observed tissue-specific effects. On the other hand, ectopic overexpression of ARNT in COS-7 cells after transfection of increasing concentrations of an ARNT expression construct did not compensate for AhRR’s inhibitory effect on DRE reporter activity (Evans et al. 2008). Interestingly, a tissue-specific regulation of AhRR on CYP1A1 induction has been reported also from AhRR null mice showing a superinduction of CYP1A1 by TCDD only in spleen, skin, and stomach, but not in other tissues of AhRR null mice (Hosoya et al. 2008). It is possible that tissue-specific co-factors are responsible for the interaction with AhRR and the subsequent suppression of CYP1A1 induction. Cofactors including Ankyrin-repeat protein2 and histone deacetylases have been reported to be involved in the inhibitory activity of the AhRR (Oshima et al. 2007).

**Figure 13:**
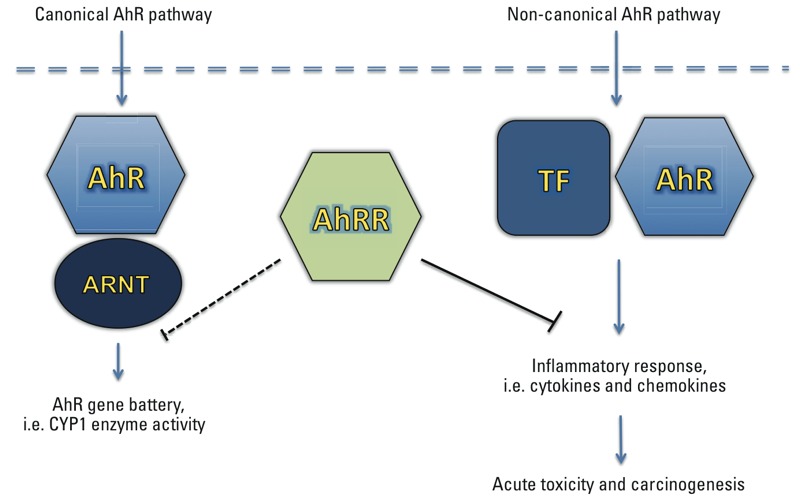
Hypothetical scheme of AhRR repression on AhR activity and the consequences in endpoints. Inhibition of the canonical AhR/ARNT pathway by AhRR can be mediated through a simple negative feedback mechanism based on its ability to form a complex with AhR’s dimerization partner ARNT. Furthermore, the AhRR can repress inflammatory genes independently from AhR/ARNT by interacting with other transcription factors (TF) such as subunits of the C/EBP and NF-κB family, which are involved in non-canonical AhR signaling.

Besides the lower TCDD-induced CYP1A1 expression in some types of tissue, we observed a constantly lower level of TCDD-induced expression of chemokines and cytokines such as IL-1β in the tissues of AhRR Tg mice compared with wt mice. Although the investigated cytokines and chemokines contain one or more DRE consensus elements on their promoter regions, the number and location of the DRE consensus sites do not necessarily correlate with the TCDD-inducibility of the specific chemokine or cytokine. One reason is that the flanking regions of the particular DRE site are playing a critical role for the functional binding of the AhR/ARNT complex as reported earlier (Yao and Denison 1992). We found evidence of tissue-specific reduced DRE binding activity and lower DNA binding activities for the transcription factors C/EBP and NF-κB in TCDD-treated AhRR Tg mice compared to TCDD-treated wt mice. This mechanism of inhibitory action by the AhRR is thought to be independent of competition for ARNT and may involve transrepression mechanism as hypothesized previously (Evans et al. 2008; Hahn et al. 2009). A mechanism of AhRR repression independent of competition for ARNT has been described also for the human AhRR (Karchner et al. 2009). The transrepression mechanism may act independently from direct DNA binding by the repressor through protein–protein interactions with other transcription factors. A possible interaction of AhRR with proteins other than those in the bHLH-PAS family is supported by a report showing a repressed ERα-mediated transactivation of reporter genes and endogenous target genes (Kanno et al. 2008). A proposed model of the AhRR-mediated repression of AhR activity is shown in Figure 13.

One possible reason for suppression of specific genes such as IL-1β or CXCL2 and CXCL3 through the overexpression of AhRR is the interaction of AhRR with yet unidentified DNA sequences on the promoter regions of these particular genes. However evidence of reduced activation of C/EBP and NF-κB in response to TCDD in AhRR Tg mice suggests inhibition of an upstream signaling event for TCDD-mediated activation of a non-canonical AhR pathway. The activation of PKA for instance has been shown to be required for TCDD’s activation of C/EBPβ (Vogel et al. 2004) and PKA is involved in the non-canonical, ligand-independent activation of the AhR signaling pathway (Oesch-Bartlomowicz et al. 2005; Vogel et al. 2007b). The activation of NF-κB by TCDD may involve AhR-dependent oxidative signals. Oxidative stress generated by TCDD-induced CYP1 activity could trigger signaling pathways like PI3K and MAPK, which may activate NF-κB (Puga et al. 2000). The activation of NF-κB through AhR signaling could also depend on interaction of AhR with RelA as shown with NF-κB response elements of the IL-6 or c-myc promoter (Kim et al. 2000; DiNatale et al. 2010; Chen et al. 2012). Additionally, the AhR may interact with NF-κB RelB to induce IL-8 and other chemokines (Vogel et al. 2007b). Furthermore, elevated levels of IL-1β and IL-6 induced by TCDD might play an important role as activator of the transcription factors NF-κB as well as C/EBP and the subsequent induction of cytokines, which are regulated by these transcription factors.

Numerous studies demonstrated that the AhR is a critical transcription factor regulating immune responses and inflammatory gene expression (Stockinger et al. 2014). The results of the current study show that TCDD induced a specific set of cytokines and CXCL chemokines in various tissues of mice. The substantially higher magnitude of CXCL chemokine and IL-1β expression in response to TCDD in epididymal adipose tissue compared with other tissues suggests that the adipose may be a primary target tissue for TCDD’s effect on cytokine induction. The highest increase of more than 1,700-fold was found for CXCL5 followed by CXCL2 and CXCL3 in adipose. CXCL2 and CXCL3 control migration and adhesion of monocytes by interacting with the chemokine surface receptor CXCR2 (Lawrence 2007). CXCL5 stimulates, like CXCL1 or IL-8, the chemotaxis of neutrophils (Chang et al. 1994). CXCL5 is expressed in highly specialized cells such as white adipose tissue macrophages (Chavey et al. 2009) and the expression of CXCL5 can be upregulated by TNFα, IL-1β, or phorbol 12-myristate 13-acetate (Chang et al. 1994). Therefore, increased production of IL-1β in TCDD-treated mice may be a contributing factor to the distinctly elevated levels of CXCL5. The results of the current study showed a rapid accumulation of CD11b+ neutrophils as well as dendritic cells and inflammatory macrophages in adipose tissue associated with a strong increase of cytokine and chemokine levels in this tissue. It is not quite clear if TCDD-induced levels of cytokines are released by recruited immune cells or if immune cells were recruited by an increased production of cytokines from residing tissue cells. However, while mean numbers of total immune cells, F4/80^+^CD11b^+^ cells, and F4/80^–^CD11b^+^ cells were slightly lower on the 3rd and 6th day after TCDD in AhRR Tg mice compared with wt mice, differences were not statistically significant (Figure 6D). One possible explanation is that we found comparable levels of CXCL5 in response to TCDD in adipose of wt and AhRR Tg mice and that CXCL5 is critical for the accumulation of F4/80^+^CD11b^+^ and F4/80^–^CD11b^+^ cells. It is interesting to note that CXCL5 seems to be involved in the promotion of obesity by inhibiting insulin signaling and insulin-induced glucose transport *in vitro* (Chavey et al. 2009) since an experimental study with mice (La Merrill et al. 2009) and observational studies of humans (Fujiyoshi et al. 2006; Warner et al. 2013) suggest that TCDD exposure may promote the development of obesity and diabetes. In contrast to TCDD-induced wasting syndrome in mice at higher doses, the reports named above showed that administration of low doses of TCDD may cause weight gain and metabolic syndrome.

Besides the suppressed expression of TCDD-induced cytokines we found a reduced acute toxicity in AhRR Tg mice indicated by lower levels of ALT and TRG in liver and an increased resistance toward lethal doses of TCDD. Although male mice of both strains wt and AhRR Tg mice lost a large amount of bodyweight, more than 50% of male AhRR Tg mice survived the lethal dose of TCDD. The observed gender difference in TCDD sensitivity of acute toxicity in mice confirms previous studies showing significantly less signs of acute toxicity in female mice compared to male mice (Pohjanvirta et al. 2012). The gender-specific difference in mice and the mechanism of the reduced lethality in AhRR Tg male mice are unclear, but might be related to a reduced systemic inflammation and tissue injury. TCDD is known to affect the function of various types of vital organs including kidney and liver, which is associated with increased inflammation (Pande et al. 2005; Nishimura et al. 2008). The importance of IL-1β in mediating TCDD’s toxic effects has been well demonstrated using “triple-null” mice lacking IL-1 and TNFα receptors, which were protected from TCDD-induced liver inflammation and inflammatory cell infiltration (Pande et al. 2005). Thus, AhRR Tg mice as well as female mice might be protected from inflammatory tissue injury and high-dose TCDD-induced toxicity by a repressed expression of inflammatory cytokines especially IL-1β. Other environmental toxicants of different structure than dioxins may also cause changes in the expression of inflammatory mediators involving macrophages as a key cell type to regulate cytokines in chemical toxicity (Laskin 2009). Furthermore, a link of environmental exposure with changes of inflammatory mediators and the possible consequences in carcinogenesis has been recently reviewed (Thompson et al. 2015).

Beyond the role as a suppressor of the AhR signaling pathway, recent reports demonstrated the potential role of the AhRR in cancer biology acting as a tumor suppressor gene (Schlezinger et al. 2006; Zudaire et al. 2008). An increased expression and activity of AhR in inflammatory disease as well as in various tumors and cancer cell lines has been reported and the critical role of AhR in tumorigenesis is well established (Vogel et al. 2007a, 2014; Murray et al. 2014). The role of the AhRR as an inhibitor of inflammatory responses could be implemented in the anti-carcinogenic action of the AhRR since IL-1β signaling and a pro-inflammatory microenvironment is well known to promote early processes of carcinogenesis (Jain et al. 2014).

## Conclusion

In summary, using a transgenic mouse model overexpressing AhRR we showed that AhRR repressed the induction of CYP1A1 by TCDD in a tissue-specific manner. Of the tissues evaluated in this study, the greatest response of TCDD to induce inflammatory cytokines was found in the white epididymal adipose tissue. Cytokine levels following TCDD were lower in AhRR Tg mice than in wt mice, consistent with suppressed activation of C/EBP and NF-κB *in vivo*. Furthermore, overexpression of AhRR protected from hepatic injury and acute toxicity of TCDD in mice. Additional studies are needed to investigate whether anti-inflammatory effects of AhRR may contribute to tumor suppression.

## Supplemental Material

(620 KB) PDF
