# Supplementary material for: Transgenic Overexpression of Aryl Hydrocarbon Receptor Repressor (AhRR) and AhR-Mediated Induction of CYP1A1, Cytokines, and Acute Toxicity
Source: Environ Health Perspect. 2016 Feb 5;124(7):1071–83. doi: 10.1289/ehp.1510194 (PMC4937866; doi:10.1289/ehp.1510194)
Supplement: (620 KB) PDF [file ehp.1510194.s001.acco.pdf]

Environ Health Perspect
DOI: 10.1289/ehp.1510194

Note to readers with disabilities: *EHP* strives to ensure that all journal content is accessible to all readers. However, some figures and Supplemental Material published in *EHP* articles may not conform to 508 standards due to the complexity of the information being presented. If you need assistance accessing journal content, please contact ehp508@niehs.nih.gov. Our staff will work with you to assess and meet your accessibility needs within 3 working days.

Supplemental Material

Transgenic Overexpression of Aryl Hydrocarbon Receptor Repressor (AhRR) and AhR-Mediated Induction of CYP1A1, Cytokines, and Acute Toxicity

Christoph F.A. Vogel, W.L. William Chang, Sarah Kado, Kelly McCulloh, Helena Vogel, Dalei Wu, Thomas Haarmann-Stemmann, GuoXiang Yang, Patrick S.C. Leung, Fumio Matsumura, and M. Eric Gershwin

Table of Contents

Supplemental Material, Table S1. Primer used to amplify mRNAs via quantitative real-time PCR encoding mouse rps13 as housekeeping gene, CYP1A1, AhRR, cytokines, and COX-2 based on published GenBank sequences for mouse.
Supplemental Material, Table S2. AhR-mediated induction of CYP1A1, cytokines, TNFα, and COX-2 in WT and AHRR Tg male mice.
Supplemental Material, Figure S1. H&E staining of various tissues from wt and AhRR Tg mice. At necropsy liver, lung, inguinal lymph nodes with surrounding white adipose tissue, thymus and spleen from wt and AhRR Tg mice were fixed in 10% formalin and then paraffin-embedded using standard histology protocols. Tissue sections represent replicates from four mice in each group control wt and AhRR Tg mice. Five-µm sections were cut and stained with H&E as described previously (Wu et al. 2012).
Supplemental Material, Figure S2. Expression of CXCL chemokines, cytokines and COX-2 in epididymal adipose tissue of AhR null mice (AhR-/-). (A) Expression of CXCL1, CXCL2, CXCL3, CXCL5, CXCL7, and CXCL14 and (B) IL-1β, IL-6, IL-10, IL-22, TNFα, and COX-2 in adipose tissue of AhR-/- mice in response to TCDD. Male AhR-/- mice were injected i.p. with a single dose of 20 µg/kg TCDD for 24h. Control

animals received the solvent vehicle. Total RNA from tissues of six mice from each group was subjected to qPCR analysis as described under Materials and Methods. Data are presented as mean ± SD. ns, not significant by two-tailed Student's t-test or Bonferroni's test.

Supplemental Material, Figure S3. Expression of AhRR, CYP1A1, and cytokines in female mice in response to TCDD. Expression of AhRR, CYP1A1, CXCL chemokines and cytokines in spleen and adipose tissue of female C57BL/6 wt and female AhRR Tg mice in response to TCDD. Female mice were injected i.p. with a single dose of 20 μg/kg TCDD for 24h. Control animals received the solvent vehicle. Total RNA from tissues of six mice from each group was subjected to qPCR analysis as described under Materials and methods. The values are given as relative units and presented as mean ± SD. [*]Significantly different from female wt control, $p < 0.05$; [**]Significantly different from female wt TCDD, $p < 0.05$. [a]Significantly different from male wt TCDD, $p < 0.05$; [b]Significantly different from male AhRR Tg TCDD, $p < 0.05$, by two-tailed Student's ***t***-test or Bonferroni's test.

Reference

Supplemental Material, Table S1. Primer used to amplify mRNAs via quantitative real-time PCR encoding mouse rps13 as housekeeping gene, CYP1A1, AhRR, cytokines, and COX-2 based on published GenBank sequences for mouse.

Gene	**Forward primer (5′–3′)**	**Reverse primer (5′–3′)**
AhRR	tggacaagctttctgtcctg	cgaagccattgagagactcc
COX-2	tttgttgagtcattcaccaga	cagtattgaggagaacagat
CXCL1	cttgaaggtgttgccctca	tggggacaccttttagcatc
CXCL2	aagtttgccttgaccctgaa	aggcacatcaggtacgatcc
CXCL3	caacggtgtctggatgtgtc	agccaaggaatactgcctca
CXCL5	gaaagctaagcggaatgcac	gggacaatggtttccctttt
CXCL7	gcgctgcagatgtacgaata	aggaaaatggtttggcacag
CXCL10	ggatggctgtcctagctctg	ataaccccttgggaagatgg
CXCL14	ctccaggccagttgagagac	ctggaagcctttcacacaca
CYP1A1	ggccactttgacccttacaa	caggtaacggaggacaggaa
IL-1β	gcccatcctctgtgactcat	aggccacaggtattttgtcg
IL-6	ccggagaggagacttcacag	ggaaattggggtaggaagga
IL-10	ccaagccttatcggaaatga	ttttcacaggggagaaatcg
IL-22	tttcctgaccaaactcagca	tctggatgttctggtcgtca
TNFα	agcccccagtctgtatcctt	ctccctttgcagaactcagg
Rps13	gtccgaaagcaccttgagag	agcagaggctgtggatgact

Supplemental Material, Table S2. AhR-mediated induction of CYP1A1, cytokines, TNFα, and COX-2 in WT and AhRR Tg male mice.

Gene	ADIPOSE AhRR Tg ctrl vs. wt ctrl	ADIPOSE wt TCDD vs. wt ctrl	ADIPOSE AhRR Tg TCDD vs. wt TCDD	KIDNEY AhRR Tg ctrl vs. wt ctrl	KIDNEY wt TCDD vs. wt ctrl	KIDNEY AhRR Tg TCDD vs. wt TCDD	LIVER AhRR Tg ctrl vs. wt ctrl	LIVER wt TCDD vs. wt ctrl	LIVER AhRR Tg TCDD vs. wt TCDD
CYP1A1	—	↑	↓	↑	↑	↓	—	↑	↑↓
CXCL1	—	↑	↓	—	↑	—	—	↑	↓
CXCL2	—	↑	↓	—	↑	↓	—	↑	↓
CXCL3	—	↑	↓	—	↑	↓	—	↑	↓
CXCL5	—	↑	↑↓	—	↑	↓	—	↑	↓
CXCL7	—	↑	↑↓	—	↑	↑↓	—	—	—
CXCL14	—	↑	↑↓	—	↓	↑↓	—	—	—
IL-1β	—	↑	↓	—	↑	↓	—	↑	↓
IL-6	—	↑	↑↓	—	↑	↑↓	—	—	—
IL-10	—	↑	↑↓	—	↑	—	—	—	—
IL-22	—	—	—	—	↑	—	—	—	—
TNFα	—	↑	↓	—	—	—	—	—	—
COX-2	—	↑	↓	—	↑	↓	—	↑	↓

Gene	LUNG AhRR Tg ctrl vs. wt ctrl	LUNG wt TCDD vs. wt ctrl	LUNG AhRR Tg TCDD vs. wt TCDD	SPLEEN AhRR Tg ctrl vs. wt ctrl	SPLEEN wt TCDD vs. wt ctrl	SPLEEN AhRR Tg TCDD vs. wt TCDD	THYMUS AhRR Tg ctrl vs. wt ctrl	THYMUS wt TCDD vs. wt ctrl	THYMUS AhRR Tg TCDD vs. wt TCDD
CYP1A1	↓	↑	↑↓	—	↑	↓	—	↑	↑↓
CXCL1	—	—	—	—	↑	↓	—	↑	↑↓
CXCL2	—	↑	↓	—	↑	↓	—	↑	↓
CXCL3	—	↑	↑↓	—	↑	↓	—	↑	↓
CXCL5	—	—	—	—	↑	↑↓	—	↑	↑↓
CXCL7	—	—	—	—	—	—	—	↑	↑↓
CXCL14	—	↓	↑↓	—	—	—	—	↑	—
IL-1β	—	↑	↓	—	↑	↓	—	↑	↓
IL-6	—	—	—	—	↑	—	—	—	—
IL-10	—	—	—	—	↑	↑↓	—	↑	↑↓
IL-22	—	—	—	—	—	—	—	↑	↑↓
TNFα	—	—	—	—	—	—	—	—	—
COX-2	—	↑	↓	—	↑	↓	—	↑	↑↓

legend:

—	not significantly different vs. wt ctrl
↑	significantly higher (AhRR Tg Ctrl vs. wt Ctrl); $p < 0.05$
↓	significantly lower (AhRR Tg Ctrl vs. wt Ctrl); $p < 0.05$
↑	significantly higher (wt TCDD vs. wt Ctrl) ; $p < 0.05$
↓	significanly lower (wt TCDD vs. wt Ctrl); $p < 0.05$
↓	significantly lower (AhRR Tg TCDD vs. wt TCDD); $p < 0.05$
↑↓	not significantly different (AhRR Tg TCDD vs. wt TCDD); $p < 0.05$

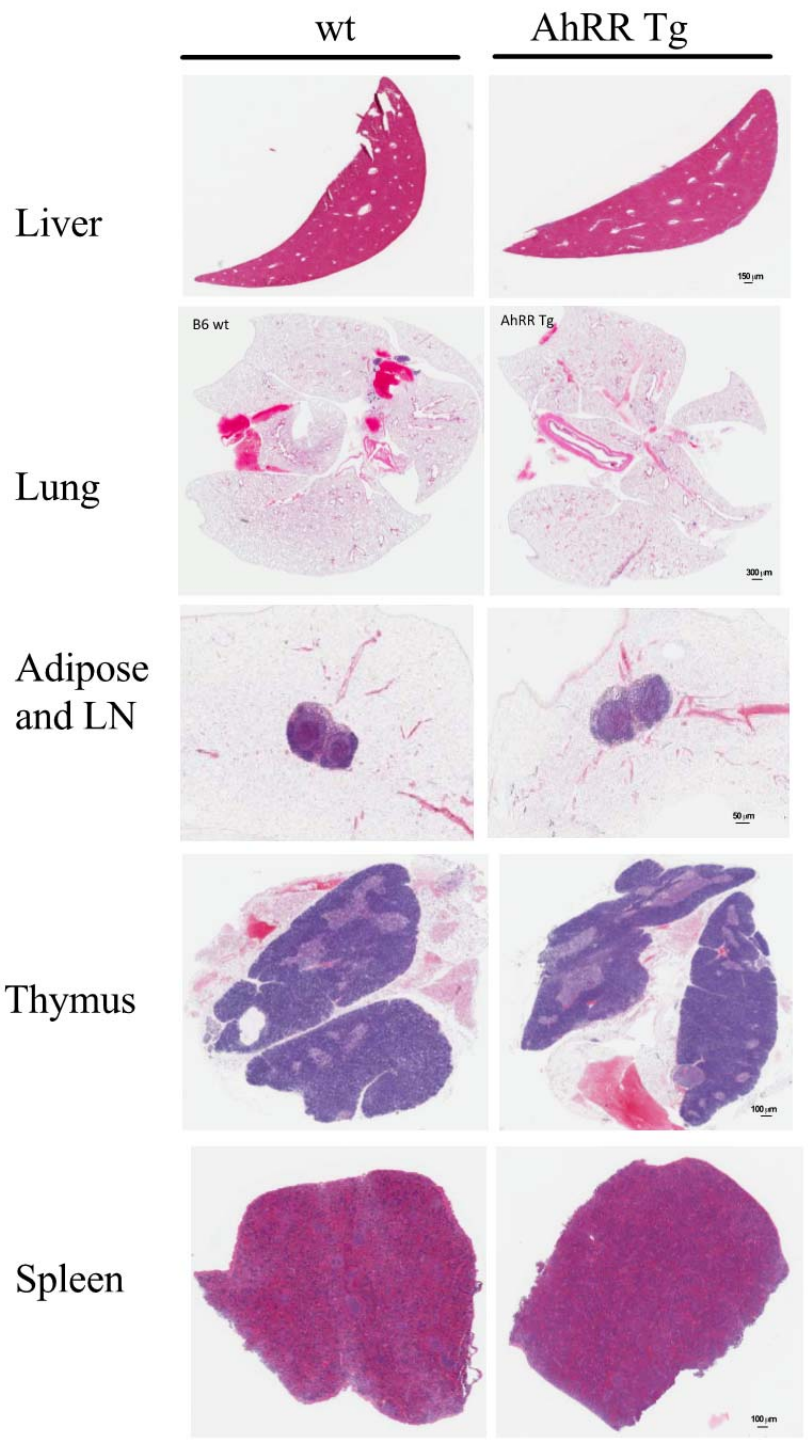

Supplemental Material, Figure S1. H&E staining of various tissues from wt and AhRR Tg mice. At necropsy liver, lung, inguinal lymph nodes with surrounding white adipose tissue, thymus and spleen from wt and AhRR Tg mice were fixed in 10% formalin and then paraffin-embedded using standard histology protocols. Tissue sections represent replicates from four mice in each group control wt and AhRR Tg mice. Five-µm sections were cut and stained with H&E as described previously (Wu et al. 2012).

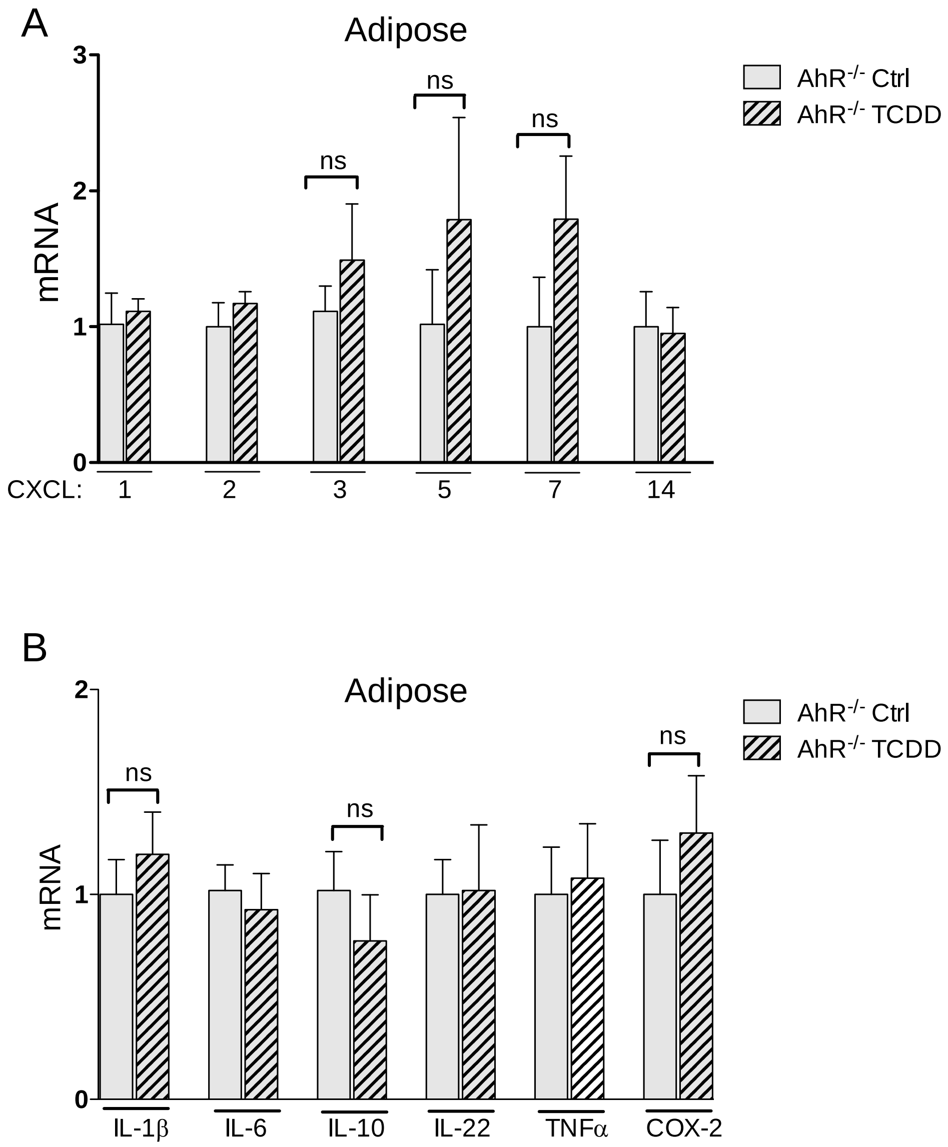

Supplemental Material, Figure S2. Expression of CXCL chemokines, cytokines and COX-2 in epididymal adipose tissue of AhR null mice ($AhR^{-/-}$). (A) Expression of CXCL1, CXCL2, CXCL3, CXCL5, CXCL7, and CXCL14 and **(B)** IL-1β, IL-6, IL-10, IL-22, TNFα, and COX-2 in adipose tissue of $AhR^{-/-}$ mice in response to TCDD. Male $AhR^{-/-}$ mice were injected i.p. with a single dose of 20 μg/kg TCDD for 24h. Control animals received the solvent vehicle. Total RNA from tissues of six mice from each group was subjected to qPCR analysis as described under Materials and Methods. Data are presented as mean ± SD. ns, not significant by two-tailed Student's ***t***-test or Bonferroni's test.

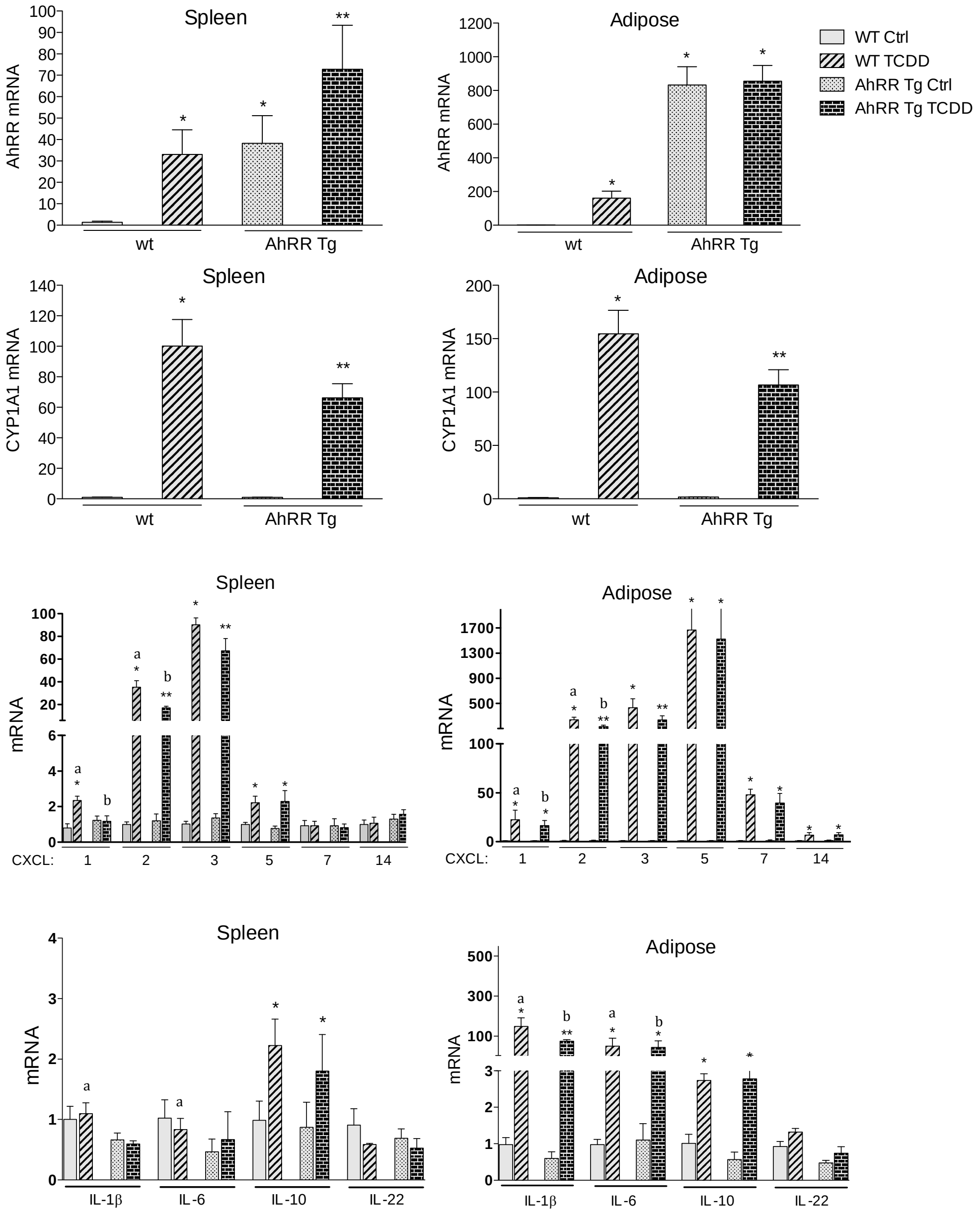

Supplemental Material, Figure S3. Expression of AhRR, CYP1A1, and cytokines in female mice in response to TCDD. Expression of AhRR, CYP1A1, CXCL chemokines and cytokines in spleen and adipose tissue of female C57BL/6 wt and female AhRR Tg mice in response to TCDD. Female mice were injected i.p. with a single dose of 20 µg/kg TCDD for 24h. Control animals received the solvent vehicle. Total RNA from tissues of six mice from each group was subjected to qPCR analysis as described under Materials and methods. The values are given as relative units and presented as mean ± SD. *Significantly different from female wt control, $p < 0.05$; **Significantly different from female wt TCDD, $p < 0.05$. ^{a}Significantly different from male wt TCDD, $p < 0.05$; ^{b}Significantly different from male AhRR Tg TCDD, $p < 0.05$, by two-tailed Student's ***t***-test or Bonferroni's test.

Reference

Wu D, Nishimura N, Kuo V, Fiehn O, Shahbaz S, Van Winkle L, et al. 2011. Activation of aryl hydrocarbon receptor induces vascular inflammation and promotes atherosclerosis in apolipoprotein E-/- mice. Arterioscler Thromb Vasc Biol 31: 1260-1267.
